# The Efficacy of Adjuvant FOLFOX6 for Patients With Gastric Cancer after D2 Lymphadenectomy: A Propensity Score-matched Analysis

**DOI:** 10.1097/MD.0000000000003214

**Published:** 2016-04-22

**Authors:** Zi-Xian Wang, Xu-Long Yang, Ming-Ming He, Feng Wang, Dong-Sheng Zhang, Yu-Hong Li, Zhi-Wei Zhou, You-Qing Zhan, Rui-Hua Xu

**Affiliations:** From the Department of Medical Oncology (Z-XW, M-MH, FW, D-SZ, Y-HL, R-HX); Faculty of Medical Sciences, (Z-XW, X-LY); and Department of Gastric and Pancreatic Surgery, Sun Yat-sen University Cancer Center, State Key Laboratory of Oncology in South China, Collaborative Innovation Center for Cancer Medicine, Guangzhou, China (Z-WZ, Y-QZ).

## Abstract

Adjuvant 5-fluorouracil, folinic acid, and oxaliplatin (FOLFOX6) are widely used for treating resected gastric cancer in clinics in China, but only few clinical trials have investigated its efficacy. Using propensity score matching, we evaluated the efficacy of adjuvant FOLFOX6 following D2 lymphadenectomy.

Patients who received adjuvant FOLFOX6 following D2 lymphadenectomy (FOLFOX6, n = 113) or D2 lymphadenectomy only (surgery-only, n = 512) between 1998 and 2007 at our center were propensity score-matched; we identified a balanced 1:2 cohort, with 96 patients in the FOLFOX6 group and 192 patients in the surgery-only group. The overall survival (OS) was estimated using the Kaplan–Meier method; factors affecting survival were identified by Cox regression models. A nomogram incorporating independent prognosticators was constructed for predicting the 3-, 5-, and 7-year OS, and bootstrap validation was performed.

The median follow-up was 9.3 years, and the 7-year OS was 52.1% in the FOLFOX6 group and 43.8% in the surgery-only group (*P* = 0.04), with an adjusted hazard ratio of 0.69 (95% confidence interval = 0.49–0.98). A prognostic nomogram was generated with the identified significant prognosticators (adjuvant FOLFOX6, number of total harvested nodes, the interaction effect between these two variables, tumor size, T and N stage). Internal validation of the nomogram revealed good predictive abilities, with a bootstrap-corrected concordance index of 0.70.

Adjuvant FOLFOX6 following D2 lymphadenectomy is associated with survival benefit in resected gastric cancer. Receiving adjuvant FOLFOX6 can be developed into a nomogram with other independent prognosticators to refine OS prediction and estimation of benefit from adjuvant FOLFOX6 for resected gastric cancer.

## Abstract 2

Supplemental Digital Content is available in the text

## INTRODUCTION

Although the incidence and mortality rate of gastric cancer have been decreasing for decades, it remains the fifth most common malignancy and the third leading cause of cancer-related death worldwide,^1^ with especially high incidence in East Asia. Radical resection remains the only possible cure for gastric cancer, especially for patients with early-stage disease.^2^ However, despite extended lymphadenectomies being performed, the high rate of postsurgical recurrence leads to dismal prognoses for patients with locally advanced tumors, suggesting the presence of micrometastatic disease at surgery.^3,4^ Based on these observations, it was hypothesized that adjuvant chemotherapy could improve outcomes in resected locally advanced gastric cancer,^5^ and two milestone meta-analyses by the GASTRIC Group and the Cochrane Collaboration confirmed this hypothesis, both demonstrating 15% to 18% reduced risk of death by adjuvant chemotherapy.^6,7^

However, the best treatment option in the adjuvant setting remains inconclusive. Generally, surgery followed by 5-fluorouracil (5-FU)-based chemoradiotherapy is the standard of care for patients with resected gastric cancer in the United States, which is primarily based on the results of the Intergroup 116 trial, in which only 10% of patients underwent D2 lymphadenectomy.^8^ For patients who have undergone D2 lymphadenectomy, the use of adjuvant chemotherapy is mainly supported by the ACTS-GC trial and the CLASSIC trial, which used adjuvant S-1 and capecitabine plus oxaliplatin (XELOX), respectively.^9,10^ In addition, several trials failed to demonstrate the benefit of more intense regimens such as sequential polychemotherapy.^11,12^

In clinics in China, adjuvant 5-FU, folinic acid (FA), and oxaliplatin (FOLFOX6) were widely used during the period in which standard protocols for adjuvant therapy were lacking.^13,14^ However, only one small prospective, randomized controlled trial investigating the efficacy of adjuvant FOLFOX (FOLFOX4) in patients with D2-resected gastric cancer was published.^15^

In the present study, we retrospectively investigated the efficacy of adjuvant FOLFOX6 in patients after D2 lymphadenectomy. Propensity score matching (PSM) was used to adjust for treatment selection bias, and a nomogram was generated to refine the prediction of prognosis and to evaluate the potential benefit from adjuvant FOLFOX6 in these patients.

## METHODS

### Ethics Statement

All of the patients provided written informed consent for the storage and use of their information in the hospital database. Study approval was obtained from the independent ethics committees at the Sun Yat-sen University Cancer Center. The study was undertaken in accordance with the ethical standards of the World Medical Association Declaration of Helsinki.

### Patient Selection

Between October 1998 and July 2007, we identified 796 patients who had been diagnosed with gastric cancer and undergone D2 lymphadenectomy at the Sun Yat-sen University Cancer Center. Patients included in the study met the following criteria: (1) aged 20 to 75 years; (2) histologically confirmed stage IB–IIIC gastric adenocarcinoma; (3) underwent histologically confirmed R0 resection, with or without adjuvant FOLFOX4/6; (4) availability of complete clinicopathologic and follow-up data. The exclusion criteria were: (1) death within 90 days of surgery; (2) age >75 years or <20 years; (3) presence of residual macroscopic or microscopic tumor, distant metastasis, or concurrent malignancies in other organs; (4) received neoadjuvant chemo/radiotherapy, adjuvant radiotherapy, or adjuvant chemotherapy regimens other than FOLFOX4/6. Eventually, a total of 625 patients were identified as the study cohort: 113 received adjuvant FOLFOX (all received FOLFOX6) and 512 underwent surgery alone.

The clinical decision to administer postoperative chemotherapy was based on the patient's disease stage, general health, and preference. All of the patients provided informed consent before receiving the adjuvant FOLFOX6. The FOLFOX6 regimen consisted of 2-week cycles of intravenous 100 mg/m^2^ oxaliplatin and 400 mg/m^2^ FA (or 200 mg/m^2^ leucovorin [LV]) over 2 h on day 1 of each cycle, plus 400 mg/m^2^ bolus 5-FU with 2400 mg/m^2^ infusional 5-FU in 46 h for each cycle. The median duration of chemotherapy was six cycles.

### Follow-up

Following treatment, patients were monitored every 3 months for the first 2 years, and then every 6 months thereafter.

### Statistical Analysis

The clinicopathologic characteristics of the cohort are described, and the differences in these characteristics between the FOLFOX6 group and surgery-only group were compared. Categorized variables were compared using the chi-square test; continuous variables were compared using the Mann–Whitney *U* test. Logistic regression analysis was used to identify confounders between the treatment groups. Propensity scores were calculated based on the identified confounders and other important factors such as tumor stage, and then each patient was assigned a score.^16^ Using 0.1-caliper width, 1:2 matching was performed between patients in the FOLFOX6 group and surgery-only group based on the propensity scores. This allowed clinical outcomes between the treatment groups to be compared without adjusting for confounders.^17^

Overall survival (OS) was calculated from the date of surgery until final follow-up or death from any cause. The Kaplan–Meier method with log-rank testing was used to assess the unadjusted survival benefit from adjuvant FOLFOX6; multivariate Cox proportional hazards regression was used to assess the survival benefit from adjuvant FOLFOX6 after adjusting for identified prognosticators and to perform interaction tests between treatment and other clinical features. The final logistic and Cox model selection was performed by stepwise forward selection: variables were added using forward selection according to a selection entry criterion of *P* < 0.05 and removed using backward elimination according to a selection stay criterion of *P* < 0.05. A nomogram for predicting individual survival was constructed based on the final Cox model. The comparative discriminative power of the nomogram and the seventh American Joint Committee on Cancer/International Union Against Cancer (AJCC/UICC) staging system was assessed using the concordance index (C-index)^18^: a higher C-index indicates more accurate prediction of prognosis.^19^ Nomogram calibration was assessed by reviewing the plot of nomogram-predicted survival probabilities versus the Kaplan–Meier-estimated probabilities.^20^ Bootstraps with 1000 resamplings were used to quantify any model overfit and to calculate the Kaplan–Meier estimates. All tests were two-sided and a *P*-value of <0.05 was considered statistically significant. Analysis was performed using SPSS version 19.0 (SPSS, Chicago, IL) and R version 3.1.2 (http://www.r-project.org/) statistical packages.

## RESULTS

### Overall Patient Characteristics

Table 1 summarizes the patient characteristics before and after PSM. Before PSM, there were significant differences in patient age, tumor location, pathologic T/N stage, and the number of total harvested lymph nodes (THN). After PSM, the confounders identified by multivariate logistic regression (age, tumor location, and T stage) and all of the other clinicopathologic factors were balanced between the treatment arms. The 1:2 propensity score–matched cohort consisted of 288 patients (96 received FOLFOX) with stage IB–IIIC gastric cancer. One hundred and ten patients (38.2%) were aged >60 years, and 129 patients (44.8%) had tumors at the distal third of the stomach. The majority of patients (233, 80.9%) had T4 lesions, and 203 patients (70.5%) had lymph node (LN) metastasis. One hundred and ninety-seven patients (68.4%) had >15 THN. The median follow-up time for the post-PSM cohort was 9.3 years (IQR, 6.2–10.2 years); specifically, 9.7 years for the FOLFOX6 group and 8.3 years for the surgery-only group. During the follow-up, 47 patients (49%) in the FOLFOX6 group and 104 patients (54%) in the surgery-only group died.

**TABLE 1:**
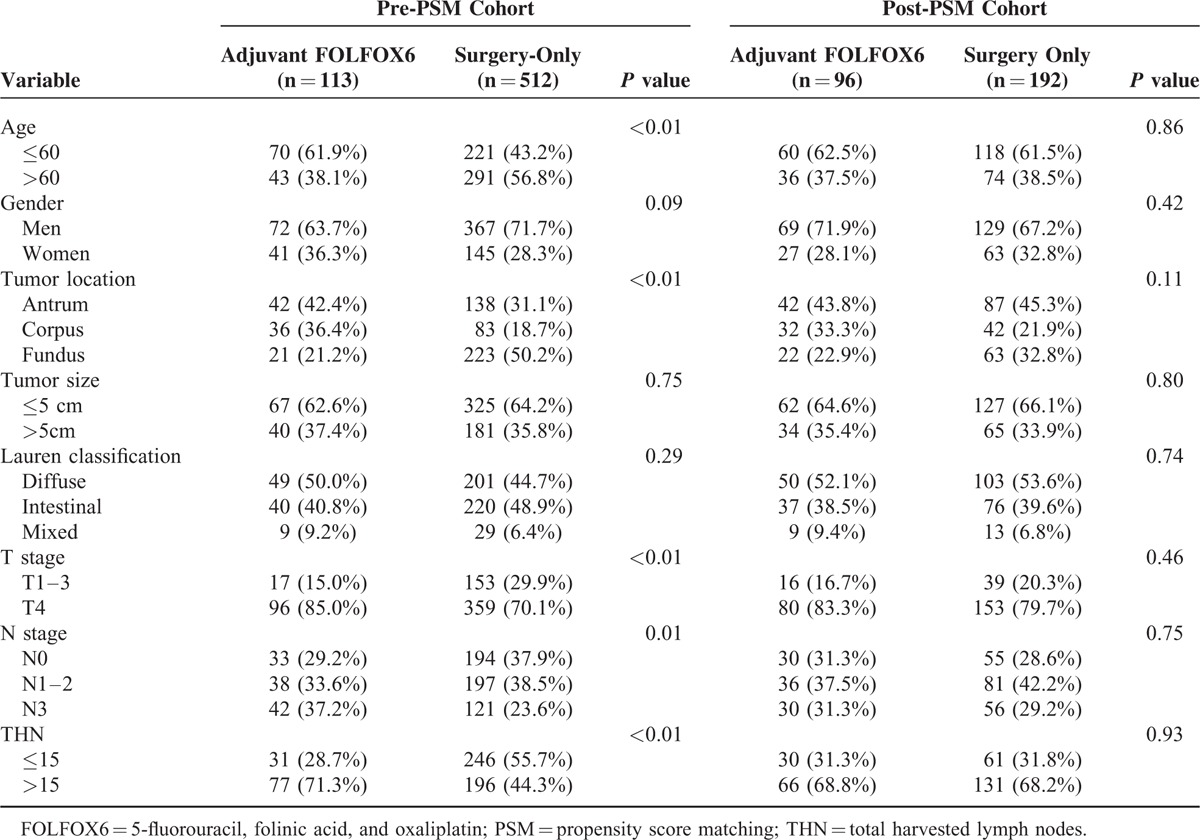
Characteristics of the Patient Cohort Before and After PSM

### Survival Analysis

**FIGURE 1:**
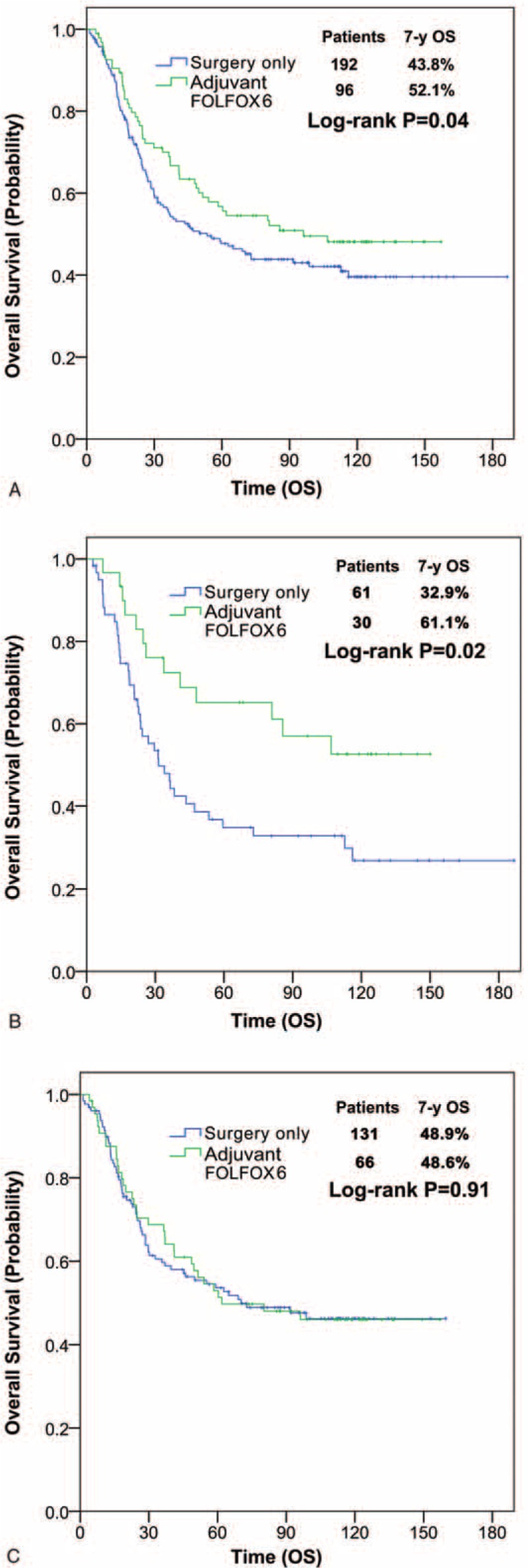
OS curves according to adjuvant FOLFOX6 treatment. (A) The entire matched cohort (n = 288); (B) patients with ≤15 THN (n = 91); (C) patients with >15 THN (n = 197). FOLFOX6 = 5-fluorouracil, folinic acid, and oxaliplatin; THN = total harvested lymph nodes.

Figure 1A depicts the OS curves for the post-PSM cohort according to treatment arm. The 3-, 5-, and 7-year OS rates were 70.0%, 56.7%, and 52.1%, respectively, in the FOLFOX6 group versus 56.0%, 45.8%, and 43.8%, respectively, in the surgery-only group (*P* = 0.04). After adjusting for tumor size, T stage, N stage, and THN, the hazard ratio (HR) for the FOLFOX6 arm as compared with the control was 0.69 (95% confidence interval [95% CI] = 0.49–0.98, *P* = 0.04), indicating that FOLFOX6 reduced the risk of death by 31% (Table 2).

**TABLE 2:**
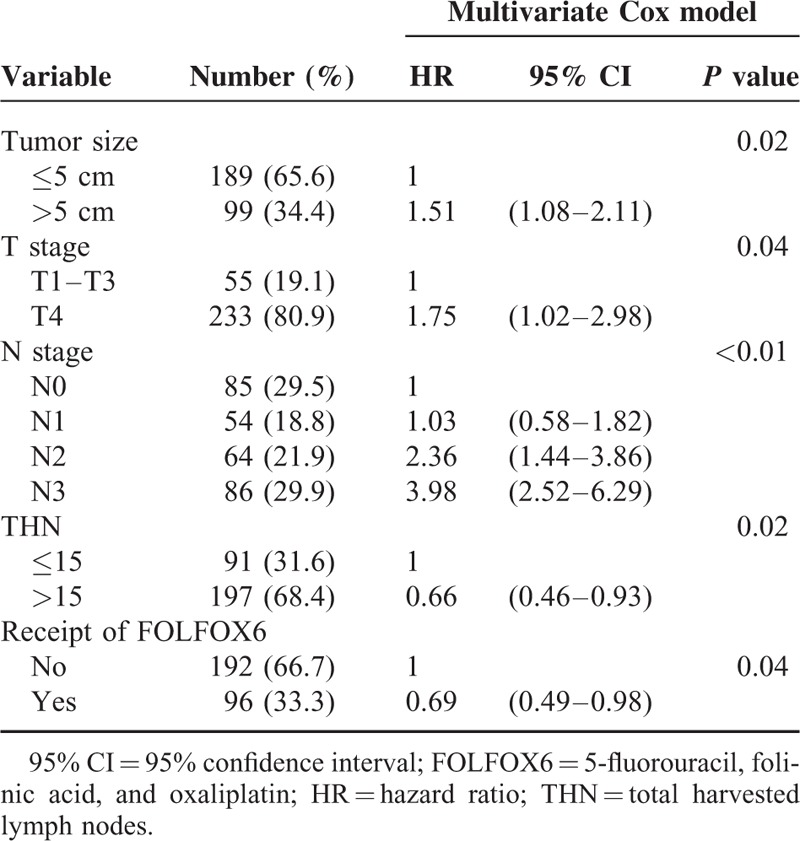
Multivariate Analysis for Identifying Prognosticators for Predicting OS

In subgroup analyses, we observed no significant interaction between treatment and any subgroup for OS, except for THN (Supplementary Table 1). Patients with ≤15 THN apparently benefited more from adjuvant FOLFOX6 as compared with those with >15 THN: the 3-, 5- and 7-year OS rates of patients with ≤15 THN were 72.4%, 65.2%, and 61.1%, respectively, in the FOLFOX6 group versus 47.9%, 34.9%, and 32.9%, respectively, in the surgery-only group (*P* = 0.02, Figure 1B), whereas that for patients with >15 THN were 67.7%, 53.6%, and 48.6%, respectively, in the FOLFOX6 group and 59.7%, 53.6%, and 48.9%, respectively, in the surgery-only group (*P* = 0.91, Figure 1C), and the interaction was significant (*P* = 0.04, Table 3). Further investigations showed that the distribution of clinical features between the treatment groups remained well balanced when stratified by ≤15 or >15 THN.

**TABLE 3:**
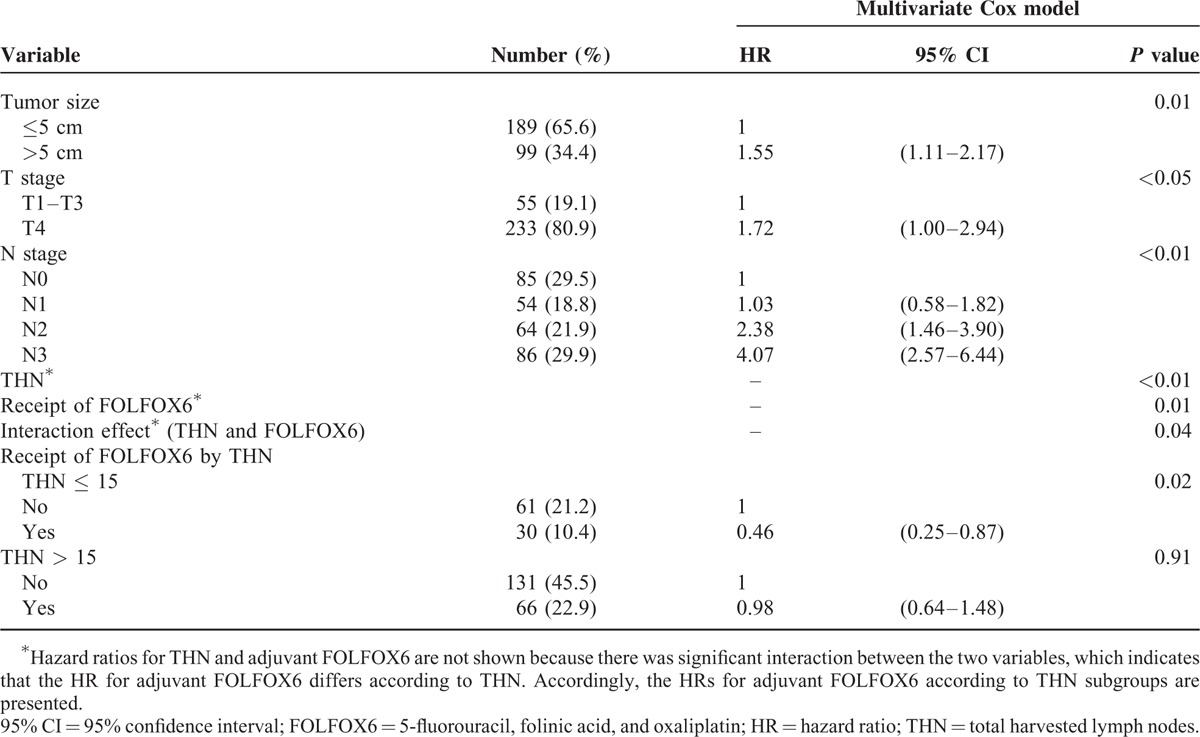
Multivariate Model Incorporating the Interaction Effect Between Adjuvant FOLFOX6 and THN for Predicting OS

### Development of Predictive Nomogram

**FIGURE 2:**
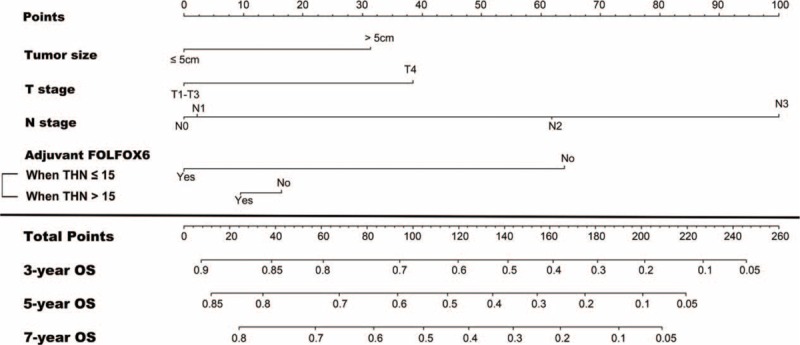
Prognostic nomogram for predicting 3-, 5-, and 7-year OS in patients with resected gastric cancer. For the factor “Adjuvant FOLFOX6 (Yes/No),” the points assigned should be chosen based on whether the patient had ≤15 or >15 THN. FOLFOX6 = 5-fluorouracil, folinic acid, and oxaliplatin; THN = total harvested lymph nodes.

**FIGURE 3:**
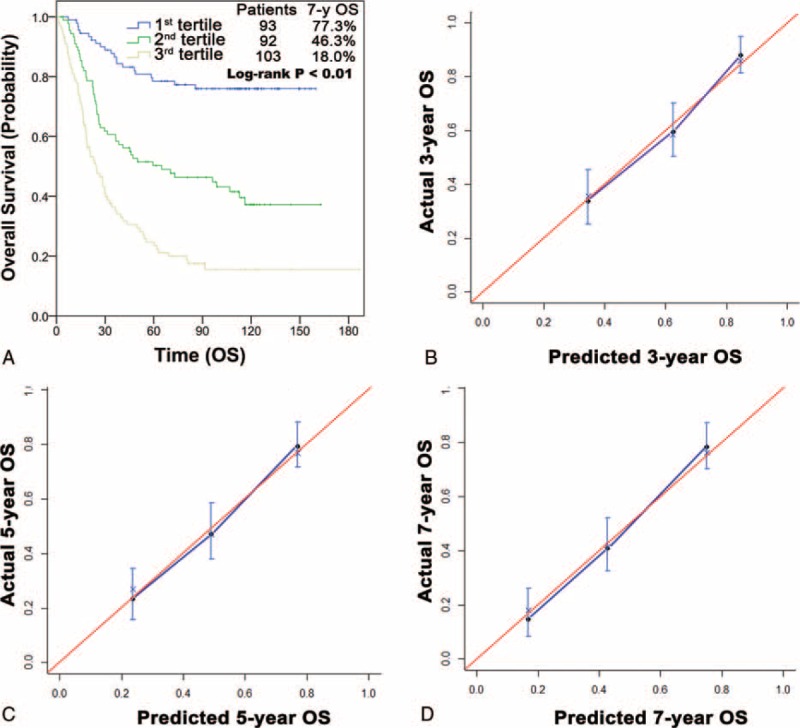
Calibration of the prognostic nomogram. (A) Kaplan–Meier curves demonstrating OS in patients grouped according to the tertiles of nomogram-predicted OS. Each group represents a distinct prognosis. Nomogram calibration plots: (B) 3-year, (C) 5-year, and (D) 7-year. The nomogram-predicted survival is closely correlated with the ideal 45° reference line. OS = overall survival.

A nomogram for predicting the 3-, 5-, and 7-year OS was generated using the multivariate analysis results (Figure 2). The independent prognosticators identified in the multivariate analysis, including tumor size, T stage, N stage, and adjuvant FOLFOX6, were incorporated into the nomogram. Moreover, because the effect size of FOLFOX6 differed according to THN, the interaction indicating the magnitude of this difference was included in the nomogram. Grouping the patients evenly into three subgroups according to the tertiles of the nomogram-calculated total scores revealed that each group represented a significantly distinct prognosis (Figure 3A).

The nomogram demonstrated good discrimination, with an unadjusted C-index of 0.71 and a 1000-sample bootstrap-corrected C-index of 0.70, which showed minimal evidence of model overfit. The nomogram had more discriminatory power than the seventh AJCC/UICC staging (unadjusted C-index, 0.67; bootstrap-corrected C-index, 0.66). Calibration plots indicated excellent predictive accuracy for the 3-, 5-, and 7-year OS, with <5% difference between the predicted and actual probabilities in each tertile (Figure 3B–D). These results all show that the nomogram performed favorably in predicting OS.

## DISCUSSION

In this study, adjuvant FOLFOX6 after D2 lymphadenectomy significantly improved the short-term and long-term survival of patients with gastric cancer as compared with surgery only, reducing the risk of death by 31%. Subset analysis suggested that adjuvant FOLFOX6 was more beneficial to patients with ≤15 THN than to those with >15 THN. To assist clinical management, the results were developed into a nomogram to predict the 3-, 5-, and 7-year OS of patients after D2 lymphadenectomy. We verified that the nomogram has good discrimination and calibration.

In addition to estimating survival probabilities, our nomogram also provides individual estimates of potential benefit from adjuvant FOLFOX6, which is helpful for management decisions. For example, a patient with stage pT4N1 (36 + 62 = 98 points) and 6-cm diameter (31 points) gastric cancer who receives adjuvant FOLFOX following surgery with a total 10 LNs harvested (0 points) would have a total score of 129 points, yielding an estimated 7-year OS of 38%. If the same patient received surgery alone, they would have a total 192 points, and the estimated 7-year OS would be only 10%, suggesting considerable benefit from adjuvant FOLFOX6 for this patient. In contrast, a patient with serosa-positive (36 points), node-negative (0 points), and 3-cm diameter (0 point) disease who receives adjuvant FOLFOX6 after D2 lymphadenectomy with a total 20 LNs harvested (10 points) would have a total 46 points and a corresponding 7-year OS of 74%. If this patient did not receive adjuvant FOLFOX6, the estimated 7-year OS would be 71%, suggesting minimal benefit from FOLFOX6 for this patient. Therefore, FOLFOX6 may be avoided when selecting an adjuvant chemotherapy regimen for this patient.

Several previous studies have failed to demonstrate the significant benefit of adjuvant chemotherapy for patients after D2 lymphadenectomy.^21–23^ Relatively good survival (range: 48%–86%) in the surgery-only groups was a common feature in these studies, which is mainly attributed to more adequate LN resection in these patients. Therefore, it was hypothesized that patients with more complete LN resection may derive less benefit from adjuvant therapy.^24^ Consistent with our results, a small, prospective, randomized controlled trial also reported that adjuvant FOLFOX (FOLFOX4) after D2 lymphadenectomy significantly improved 3-year survival outcomes for resected gastric cancer (median THN = 19) as compared with adjuvant 5-FU/LV.^15^ However, it was unclear whether the favorable outcomes in both treatment arms were because of D2 resection or adjuvant chemotherapy, and further exploratory analysis was impeded because of the small sample size.^15^

The CLASSIC trial is one of the landmark studies that confirmed the efficacy of adjuvant therapy in patients after D2 lymphadenectomy.^10^ Significantly improved 5-year disease-free survival and 5-year OS was achieved with 6 months of adjuvant XELOX. The effect size of adjuvant chemotherapy in our study was similar as compared with the CLASSIC trial (adjusted HR = 0.69 vs. 0.64), although the 5-year OS was better in both the treatment and control arms of the CLASSIC trial than in our study (treatment arm: 78% vs. 57%; control arm: 69% vs. 46%). One possible explanation is the different distribution of the clinicopathologic features between the two studies: the average THN was much higher in the CLASSIC trial than in our study (45.0 vs. 20.2), and the proportion of patients with T4 disease was much greater in our study than in the CLASSIC trial (81% vs. 44%). In addition, as a randomized controlled trial, the CLASSIC study had strict inclusion criteria, and the selected patients were in relatively good health. Therefore, the comparison of treatment efficacy between our study and the CLASSIC trial should be viewed with caution.

As it is unclear whether adjuvant XELOX outperforms adjuvant FOLFOX6, two retrospective Chinese studies compared the efficacy of FOLFOX6 with XELOX in the adjuvant setting after D2 lymphadenectomy.^13,14^ Through PSM, Wu et al observed no significant difference in efficacy between adjuvant XELOX and FOLFOX6 and no significant difference of incidence of grade 3/4 adverse effects except for more common hand–foot syndrome in the XELOX arm.^13^ The study by Chen et al^14^ from our cancer center also derived a similar conclusion for patients who had undergone total gastrectomy: who are usually diagnosed with late-stage disease and less tolerance to digestive tract toxicity.^25^ Despite the higher rate of treatment completion in the XELOX group, the 1-, 3-, and 5-year OS did not differ significantly between the treatment arms, and the incidence of grade 3/4 adverse effects was not significantly different between the treatment arms, except hand–foot syndrome was more common in the XELOX group.^14^ In addition, the M66001 trial found that the efficacy of adjuvant single-agent capecitabine and 5-FU/FA was similar in patients with resected colon cancer.^26^ All these findings suggest that FOLFOX may be an equivalent and well-tolerated alternative regimen to XELOX in the adjuvant setting for patients with gastric cancer. However, in view of the small sample size of patients receiving FOLFOX in the studies by Wu et al and Chen et al, further research on this issue is warranted.

The present study has some limitations. First, it is based on retrospective data, and there may have been treatment selection bias. Although PSM and multivariate regression were used to reduce this bias, some unaccounted confounders could still have existed between the treatment groups because of the retrospective nature and small sample size of this study. Therefore, a randomized trial or larger sample size is needed to confirm conclusions of this study. Second, we did not collect the data on treatment compliance and adverse effects for the FOLFOX6 group. However, FOLFOX4/6 has shown good tolerability in the adjuvant setting.^13,15^ In addition, the long follow-up period (median: 9.3 years) in our study could help us gain insight into the combined effect of benefit and toxicity from FOLFOX6 on long-term survival.^27^ Third, as with any predictive model, the point estimates in our nomogram might have an increased uncertainty range when applied to patients who do not have the clinicopathologic features typical of those used to generate the present nomogram.^20^ Therefore, external validation is needed. However, the 1000-sample bootstrap-corrected C-index of 0.70 for our nomogram suggests sufficient predictive accuracy and is comparable to the C-indices reported in previous nomograms (range: 0.68–0.80).^28–33^

In conclusion, we demonstrate that adjuvant FOLFOX6 therapy is associated with short-term and long-term survival benefit for patients with gastric cancer after D2 lymphadenectomy, especially for those with ≤15 LNs harvested. The results were developed into a nomogram to refine the prediction of OS for patients with resected gastric cancer. The nomogram was verified for discrimination and calibration, and internally validated by bootstrap resampling. It may be a useful tool in prognosis and treatment programming if externally validated.

## Supplementary Material

Supplemental Digital Content
