# Supplementary material for: The Efficacy of Adjuvant FOLFOX6 for Patients With Gastric Cancer after D2 Lymphadenectomy: A Propensity Score-matched Analysis
Source: Medicine (Baltimore). 2016 Apr 22;95(16):e3214. doi: 10.1097/MD.0000000000003214 (PMC4845815; doi:10.1097/MD.0000000000003214)
Supplement: Supplemental Digital Content [file medi-95-e3214-s001.doc]

**Supplementary Table 1. Subgroup analyses of the efficacy of adjuvant FOLFOX6.**

| **Variables** | **Number (%)** | **HR** | **95% CI** | ***P_interaction_^*^*** |
| --- | --- | --- | --- | --- |
| **Age** | | |  | 0.17 |
| ≤ 60 years | 178 (61.8) | 0.61 | (0.38-0.99) |  |
| > 60 years | 110 (38.2) | 1.03 | (0.62-1.70) |  |
| **Gender** | | |  | 0.60 |
| Male | 198 (68.8) | 0.73 | (0.48-1.11) |  |
| Female | 90 (31.2) | 0.87 | (0.48-1.58) |  |
| **Tumor location** | | | | 0.14 |
| Antrum | 129 (44.8) | 1.14 | (0.68-1.91) |  |
| Corpus | 74 (25.7) | 0.7 | (0.36-1.37) |  |
| Fundus | 85 (29.5) | 0.5 | (0.25-1.00) |  |
| **Tumor size** | | | | 0.92 |
| ≤ 5 cm | 189 (65.6) | 0.73 | (0.46-1.16) |  |
| > 5 cm | 99 (34.4) | 0.78 | (0.46-1.32) |  |
| **Lauren classification** | | | | 0.68 |
| Diffuse | 153 (53.1) | 0.69 | (0.43-1.11) |  |
| Intestinal | 113 (39.2) | 0.94 | (0.55-1.61) |  |
| Mixed | 22 (7.6) | 0.62 | (0.15-2.50) |  |
| **THN** | | | | **0.04** |
| ≤ 15 | 91 (31.6) | 0.46 | (0.25-0.87) |  |
| > 15 | 197 (68.4) | 0.98 | (0.64-1.48) |  |
| **T stage** | | | | 0.67 |
| T1-T3 | 55 (19.1) | 0.63 | (0.20-1.96) |  |
| T4 | 233 (80.9) | 0.76 | (0.53-1.10) |  |
| **N stage** | | | | 0.74 |
| N0 | 85 (29.5) | 0.89 | (0.41-1.92) |  |
| N1 | 54 (18.8) | 0.78 | (0.31-2.41) |  |
| N2 | 63 (21.9) | 0.50 | (0.21-1.18) |  |
| N3 | 86 (29.9) | 0.76 | (0.45-1.27) |  |

FOLFOX6, 5-fluorouracil, folinic acid, and oxaliplatin; 95% CI, 95% confidence interval; HR, hazard ratio; THN, total harvested lymph nodes.

^*^ A bold *P* value indicated statistically significant interaction.
